# National Trends in Hepatitis C Infection by Opioid Use Disorder Status Among Pregnant Women at Delivery Hospitalization — United States, 2000–2015

**DOI:** 10.15585/mmwr.mm6839a1

**Published:** 2019-10-04

**Authors:** Jean Y. Ko, Sarah C. Haight, Sarah F. Schillie, Michele K. Bohm, Patricia M. Dietz

**Affiliations:** ^1^Division of Reproductive Health, National Center for Chronic Disease Prevention and Health Promotion, CDC; ^2^National Center for HIV/AIDS, Viral Hepatitis, STD, and TB Prevention, CDC; ^3^Division of Unintentional Injury Prevention, National Center for Injury Prevention and Control, CDC.

Hepatitis C virus (HCV) is transmitted primarily through parenteral exposures to infectious blood or body fluids that contain blood (e.g., via injection drug use, needle stick injuries) (*1*). In the last 10 years, increases in HCV infection in the general U.S. population (*1*) and among pregnant women (*2*) are attributed to a surge in injection drug use associated with the opioid crisis. Opioid use disorders among pregnant women have increased (*3*), and approximately 68% of pregnant women with HCV infection have opioid use disorder (*4*). National trends in HCV infection among pregnant women by opioid use disorder status have not been reported to date. CDC analyzed hospital discharge data from the 2000–2015 Healthcare Cost and Utilization Project (HCUP) to determine whether HCV infection trends differ by opioid use disorder status at delivery. During this period, the national rate of HCV infection among women giving birth increased >400%, from 0.8 to 4.1 per 1,000 deliveries. Among women with opioid use disorder, rates of HCV infection increased 148%, from 87.4 to 216.9 per 1,000 deliveries, and among those without opioid use disorder, rates increased 271%, although the rates in this group were much lower, increasing from 0.7 to 2.6 per 1,000 deliveries. These findings align with prior ecological data linking hepatitis C increases with the opioid crisis (*2*). Treatment of opioid use disorder should include screening and referral for related conditions such as HCV infection.

To evaluate HCV infection prevalence at hospital delivery among women with and without opioid use disorder, data from HCUP’s National Inpatient Sample (NIS, 2000–2015) (https://www.hcup-us.ahrq.gov/) were analyzed. The fourth quarter of 2015 and more recent data were excluded because of the transition to the *International Classification of Diseases, Tenth Revision, Clinical Modification* (ICD-10-CM) during that period. The NIS is the largest publicly available all-payer inpatient health care database in the United States, yielding national estimates representing approximately 35 million hospitalizations. Discharges for in-hospital deliveries were identified using *International Classification of Diseases, Ninth Revision, Clinical Modification* (ICD-9-CM) diagnostic and procedure codes pertaining to obstetric delivery (*5*).

HCV infection was identified from ICD-9-CM codes 070.41, 070.44, 070.51, 070.54, 070.70, 070.71, and V02.62; and opioid use disorder was identified from codes for opioid dependence and nondependent abuse (304.00–304.03, 304.70–304.73, and 305.50–305.53), aligning with *Diagnostic and Statistical Manual of Mental Disorders, 5th Edition* criteria* (*6*). Deliveries were categorized by maternal diagnoses: HCV infection only, opioid use disorder only, both HCV infection and opioid use disorder, or neither. Demographic variables of interest included age, payer source, race/ethnicity, median income quartiles for residency ZIP code, and hospital geographic region.

Survey-specific analysis techniques accounted for clustering, stratification, and weighting. National annual prevalence rates of opioid use disorder and HCV infection per 1,000 delivery hospitalizations during 2000–2015 and 95% confidence intervals (CIs) were calculated using SAS (version 9.4; SAS Institute). HCV infection rates were calculated by opioid use disorder status. Joinpoint regression was used to model the average percentage change in HCV infection and opioid use disorder rates over time and their statistical significance. The program identifies points (joinpoints) where the slope of the trend significantly changes and calculates the average percentage change in the rate during the years between joinpoints. Using 2015 data, distribution of diagnoses by payer source, race/ethnicity, median income for residency ZIP code, and hospital region were calculated. Polytomous logistic regression models were used to calculate unadjusted odds ratios (ORs) and 95% CIs comparing the likelihood of each delivery hospitalization having one or both diagnoses versus neither by sociodemographic characteristics. Statistical significance was set at p<0.05.

During 2000–2015, the rate of HCV infection increased from 0.8 (95% CI = 0.7–0.9) to 4.1 (95% CI = 3.7–4.4) per 1,000 deliveries. Rates significantly increased from 2000 to 2004 (15.7%; p<0.001), 2004 to 2010 (6.1%; p<0.001), and 2010 to 2015 (14.9%; p<0.001). Among deliveries with opioid use disorder diagnoses, the rate of maternal HCV infection increased from 87.4 (95% CI = 56.3–118.5) to 216.9 (95% CI = 197.9–235.9) per 1,000 deliveries (Figure). The rate significantly increased during 2000–2004 (17.2%; p<0.001), remained statistically unchanged during 2004–2011 (−2.4%; p = 0.1), and significantly increased during 2011–2015 (7.9%; p<0.001). Among deliveries without opioid use disorder diagnoses, the rate of HCV infection increased from 0.7 (95% CI = 0.6–0.8) to 2.6 (95% CI = 2.4–2.9) per 1,000 deliveries during 2000–2015. The rate remained statistically unchanged during 2000–2002 (21.1%; p = 0.1), and significantly increased during 2002–2011 (5.5%; p<0.001) and 2011–2015 (15.0%; p<0.001).

**FIGURE:**
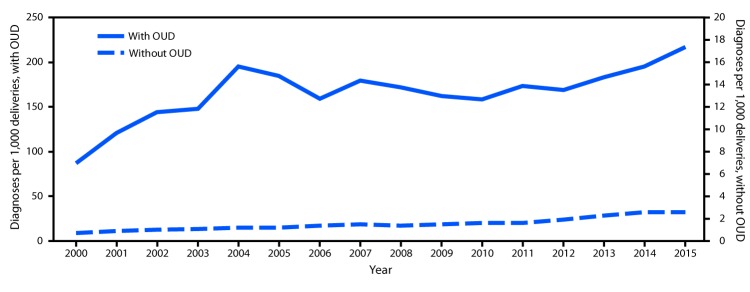
National prevalence* of maternal hepatitis C virus (HCV) infection per 1,000 delivery hospitalizations, by opioid use disorder (OUD) status, 2000–2015^†^ * Prevalence numerator consisted of HCV infection *International Classification of Diseases, Ninth Revision, Clinical Modification* (ICD-9-CM) codes (070.41, 070.44, 070.51, 070.54, 070.70, 070.71, and V02.62), and denominator consisted of delivery hospitalizations discharges with and without opioid type dependence and nondependent opioid abuse based on ICD-9-CM codes (304.00–304.03, 304.70–304.73, and 305.50–305.53). ^†^ Rates are for 2000 through the third quarter of 2015.

In 2015, all three groups (those with HCV infection only, opioid use disorder only, and both HCV infection and opioid use disorder) shared similar risk factors (Table 1). Compared with women aged ≥35 years, those aged 25–34 years were more likely to have a diagnosis of HCV infection (OR = 1.2, 95% CI = 1.0–1.4), opioid use disorder (OR = 1.8, 95% CI = 1.6–2.0), or both (OR = 1.8, 95% CI: 1.4–2.3) at delivery (Table 2). Women with publicly billed deliveries (Medicaid or Medicare) were the most likely to have a diagnosis of HCV infection (OR = 5.5, 95% CI = 4.7–6.4), opioid use disorder (OR = 6.4, 95% CI = 5.8–7.2), or both (OR = 9.9, 95% CI = 7.8–12.6) at delivery, compared with privately billed deliveries. Compared with non-Hispanic black women, Native American women were the most likely to have a diagnosis of HCV infection (OR = 5.0, 95% CI = 2.9–8.7) or opioid use disorder (OR = 5.9, 95% CI = 4.0–8.8) at delivery, and non-Hispanic white women were the most likely to have a diagnosis of both (OR = 10.9, 95% CI = 6.3–18.6) at delivery. Women from areas with median income of <$42,000 were the most likely to receive a diagnosis of HCV infection (OR = 2.5, 95% CI = 2.0–3.0), opioid use disorder (OR = 2.0, 95% CI = 1.7–2.3), or both (OR = 2.5, 95% CI = 1.8–3.4) at delivery, compared with those from areas with median income ≥$68,000. Compared with U.S. residents of the Western census region (the referent group), residents of the South were the most likely to receive a diagnosis of HCV infection (OR = 1.9, 95% CI = 1.5–2.3) at delivery. Women living in the Northeast were the most likely to receive a diagnosis of opioid use disorder (OR = 2.0, 95% CI = 1.6–2.4) or both HCV infection and opioid use disorder (OR = 4.8, 95% CI = 3.1–7.5) at delivery.

**TABLE 1:** Prevalence of hepatitis C virus (HCV) infection and opioid use disorder* at delivery hospitalization, by demographic characteristic (N = 2,860,130) — United States, 2015^†^

Characteristic	Total^§^	HCV infection only		Opioid use disorder only		HCV infection and opioid use disorder	
	No. (95% CI)	No. (95% CI)	Prevalence % (95% CI)	No. (95% CI)	Prevalence % (95% CI)	No. (95% CI)	Prevalence % (95% CI)
**Age group (yrs)**							
<25	784,830 (759,112–810,548)	1,820 (1,563–2,077)	0.2 (0.2–0.3)	4,000 (3,640–4,360)	0.5 (0.5–0.6)	1,005 (821–1,189)	0.1 (0.1–0.2)
25–34	1,616,900 (1,560,018–1,673,782)	4,560 (4,161–4,959)	0.3 (0.3–0.3)	9,380 (8,686–10,074)	0.6 (0.5–0.6)	2,695 (2,313–3,077)	0.2 (0.1–0.2)
≥35	458,380 (437,269–479,491)	1,115 (962–1,268)	0.2 (0.2–0.3)	1,495 (1,310–1,680)	0.3 (0.3–0.4)	420 (322–518)	0.1 (0.1–0.1)
**Payer source**							
Public^¶^	1,240,210 (1,193,733–1,286,686)	5,885 (5,344–6,426)	0.5 (0.4–0.5)	12,025 (11,147–12,903)	1.0 (0.9–1.0)	3,565 (3,067–4,063)	0.3 (0.2–0.3)
Private**	1,466,650 (1,401,828–1,531,472)	1,290 (1,115–1,465)	0.1 (0.1–0.1)	2,245 (1,999–2,491)	0.2 (0.1–0.2)	430 (327–533)	0.0 (0.0–0.0)
Other/Self pay^††^	148,680 (138,378–158,982)	310 (231–389)	0.2 (0.2–0.3)	575 (463–687)	0.4 (0.3–0.5)	115 (64–166)	0.1 (0.0–0.1)
**Race/Ethnicity^§§^**							
White	1,418,351 (1,362,897–1,473,804)	5,705 (5,158–6,252)	0.4 (0.4–0.4)	11,565 (10,700–12,430)	0.8 (0.8–0.9)	3,470 (2,985–3,955)	0.2 (0.2–0.3)
Black	395,535 (371,201–419,868)	450 (351–549)	0.1 (0.1–0.1)	885 (726–1,044)	0.2 (0.2–0.3)	90 (40–140)	0.0 (0.0–0.0)
Hispanic	552,715 (516,126–589,304)	470 (375–565)	0.1 (0.1–0.1)	925 (757–1,093)	0.2 (0.1–0.2)	220 (115–325)	0.0 (0.0–0.1)
Native American	19,555 (16,288–22,822)	110 (47–173)	0.6 (0.3–0.8)	255 (157–353)	1.3 (0.8–1.8)	35 (0–70)	0.2 (0.0–0.3)
Asian-Pacific Islander/Other	274,615 (252,818–296,412)	300 (206–394)	0.1 (0.1–0.1)	350 (250–450)	0.1 (0.1–0.2)	65 (1–129)	0.0 (0.0–0.0)
**Median income for ZIP code^¶¶^ ($)**							
1–41,999	822,850 (783,465–862,234)	2,935 (2,552–3,318)	0.4 (0.3–0.4)	5,225 (4,697–5,753)	0.6 (0.6–0.7)	1,630 (1,352–1,908)	0.2 (0.2–0.2)
42,000–51,999	671,335 (643,392–699,278)	2,010 (1,780–2,240)	0.3 (0.3–0.3)	3,925 (3,538–4,312)	0.6 (0.5–0.6)	1,045 (845–1,245)	0.2 (0.1–0.2)
52,000–67,999	700,610 (669,764–731,456)	1,420 (1,229–1,611)	0.2 (0.2–0.2)	3,395 (3,043–3,747)	0.5 (0.4–0.5)	840 (686–994)	0.1 (0.1–0.1)
≥68,000	628,510 (581,576–675,444)	920 (770–1,070)	0.1 (0.1–0.2)	2,050 (1,766–2,334)	0.3 (0.3–0.4)	505 (370–640)	0.1 (0.1–0.1)
Region***							
Northeast	457,160 (418,652–495,668)	1,110 (927–1,293)	0.2 (0.2–0.3)	3,390 (2,902–3,878)	0.7 (0.6–0.8)	1,190 (900–1,480)	0.3 (0.2–0.3)
Midwest	608,746 (570,546–646,947)	1,375 (1,152–1,598)	0.2 (0.2–0.3)	3,300 (2,849–3,751)	0.5 (0.5–0.6)	895 (630–1,160)	0.1 (0.1–0.2)
South	1,111,188 (1,046,643–1,175,733)	3,760 (3,265–4,255)	0.3 (0.3–0.4)	5,600 (4,941–6,259)	0.5 (0.4–0.6)	1,665 (1,313–2,017)	0.1 (0.1–0.2)
West	683,036 (637,875–728,198	1,250 (1,063–1,437)	0.2 (0.2–0.2)	2,585 (2,199–2,971)	0.4 (0.3–0.4)	370 (232–508)	0.1 (0.0–0.1)

**Abbreviation:** CI = confidence interval.

* Includes *International Classification of Diseases, Ninth Revision, Clinical Modification* codes for HCV infection (070.41, 070.44, 070.51, 070.54, 070.70–070.71, and V02.62) and opioid use disorder (304.00–304.03, 304.70–304.73, and 305.50–305.53).

^†^ Only representative of the first three quarters of 2015.

^§^ Includes deliveries with HCV infection only, opioid use disorder only, HCV infection and opioid use disorder, and neither HCV or opioid use disorder diagnoses.

^¶^ Includes Medicare and Medicaid.

** Includes Blue Cross, commercial carriers, private health maintenance organizations, and preferred provider organizations.

^††^ Includes worker’s compensation, Civilian Health and Medical Program of the Uniformed Services, Civilian Health and Medical Program of the Department of Veteran's Affairs, Title V, and other government programs.

^§§^ Whites, blacks, Native Americans, and Asian-Pacific Islanders/Others were non-Hispanic; Hispanic persons could be of any race.

^¶¶^ Estimated median household income of residents in the patient’s ZIP code derived from ZIP code demographic data obtained from Claritas (https://www.hcup-us.ahrq.gov/db/vars/zipinc_qrtl/nisnote.jsp).

*** *Northeast*: Connecticut, Maine, Massachusetts, New Hampshire, New Jersey, New York, Pennsylvania, Rhode Island, and Vermont. *Midwest*: Illinois, Indiana, Iowa, Kansas, Michigan, Minnesota, Missouri, Nebraska, North Dakota, Ohio, South Dakota, and Wisconsin. *South*: Alabama, Arkansas, Delaware, District of Columbia, Florida, Georgia, Kentucky, Louisiana, Maryland, Mississippi, North Carolina, Oklahoma, South Carolina, Tennessee, Texas, Virginia, and West Virginia. *West*: Alaska, Arizona, California, Colorado, Hawaii, Idaho, Montana, Nevada, New Mexico, Oregon, Utah, Washington, and Wyoming.

**TABLE 2:** Association of hepatitis C virus (HCV) infection and opioid use disorder* at delivery hospitalization with demographic characteristics (N = 2,860,130) — United States, 2015^†^

Characteristic	OR (95% CI)		
	HCV infection only	Opioid use disorder only	HCV infection and opioid use disorder
**Age group (yrs)**			
<25	1.0 (0.8–1.1)	1.6 (1.4–1.8)^§^	1.4 (1.1–1.8)^§^
25–34	1.2 (1.0–1.4)^§^	1.8 (1.6–2.0)^§^	1.8 (1.4–2.3)^§^
≥35	Ref.	Ref.	Ref.
**Payer source**			
Public^¶^	5.5 (4.7–6.4)^§^	6.4 (5.8–7.2)^§^	9.9 (7.8–12.6)^§^
Private**	Ref.	Ref.	Ref.
Other/Self pay^††^	2.4 (1.8–3.2)^§^	2.5 (2.0–3.1)^§^	2.6 (1.6–4.3)^§^
**Race/Ethnicity^§§^**			
White	3.6 (2.9–4.5)^§^	3.7 (3.1–4.4)^§^	10.9 (6.3–18.6)^§^
Black	Ref.	Ref.	Ref.
Hispanic	0.7 (0.6–1.0)	0.7 (0.6–1.0)	1.7 (0.8–3.6)
Native American	5.0 (2.9–8.7)^§^	5.9 (4.0–8.8)^§^	8.0 (2.7–23.5)^§^
Asian-Pacific Islander/Other	1.0 (0.7–1.4)	0.6 (0.4–0.8)^§^	1.0 (0.4–2.9)
**Median income for ZIP code^¶¶^ ($)**			
1–41,999	2.5 (2.0–3.0)^§^	2.0 (1.7–2.3)^§^	2.5 (1.8–3.4)^§^
42,000–51,999	2.1 (1.7–2.5)^§^	1.8 (1.5–2.1)^§^	1.9 (1.5–2.6)^§^
52,000–67,999	1.4 (1.1–1.7)^§^	1.5 (1.3–1.7)^§^	1.5 (1.1–2.0)^§^
≥68,000	Ref.	Ref.	Ref.
Region***			
Northeast	1.3 (1.1–1.7)^§^	2.0 (1.6–2.4)^§^	4.8 (3.1–7.5)^§^
Midwest	1.2 (1.0–1.5)	1.4 (1.2–1.8)^§^	2.7 (1.7–4.4)^§^
South	1.9 (1.5–2.3)^§^	1.3 (1.1–1.6)^§^	2.8 (1.8–4.3)^§^
West	Ref.	Ref.	Ref.

**Abbreviations:** CI = confidence interval; Ref. = referent; OR = odds ratio.

* Includes *International Classification of Diseases, Ninth Revision, Clinical Modification* codes for HCV infection (070.41, 070.44, 070.51, 070.54, 070.70–070.71, and V02.62) and opioid use disorder (304.00–304.03, 304.70–304.73, and 305.50–305.53).

^†^ Only representative of the first three quarters of 2015.

^§^ p<0.05.

^¶^ Includes Medicare and Medicaid.

** Includes Blue Cross, commercial carriers, private health maintenance organizations, and preferred provider organizations.

^††^ Includes worker’s compensation, Civilian Health and Medical Program of the Uniformed Services, Civilian Health and Medical Program of the Department of Veteran's Affairs, Title V, and other government programs.

^§§^ Whites, blacks, Native Americans, and Asian-Pacific Islanders/Others were non-Hispanic; Hispanic persons could be of any race.

^¶¶^ Estimated median household income of residents in the patient’s ZIP code derived from ZIP code demographic data obtained from Claritas (https://www.hcup-us.ahrq.gov/db/vars/zipinc_qrtl/nisnote.jsp).

*** *Northeast*: Connecticut, Maine, Massachusetts, New Hampshire, New Jersey, New York, Pennsylvania, Rhode Island, and Vermont. *Midwest*: Illinois, Indiana, Iowa, Kansas, Michigan, Minnesota, Missouri, Nebraska, North Dakota, Ohio, South Dakota, and Wisconsin. *South*: Alabama, Arkansas, Delaware, District of Columbia, Florida, Georgia, Kentucky, Louisiana, Maryland, Mississippi, North Carolina, Oklahoma, South Carolina, Tennessee, Texas, Virginia, and West Virginia. *West*: Alaska, Arizona, California, Colorado, Hawaii, Idaho, Montana, Nevada, New Mexico, Oregon, Utah, Washington, and Wyoming.

## Discussion

In the United States, the 2015 rate of HCV infection at delivery hospitalization (4.1 per 1,000) was approximately five times higher than it was in 2000 (0.8 per 1,000). Rates were substantially higher among women with opioid use disorder, suggesting a link between the opioid crisis and increases in HCV infection. Results from this analysis are consistent with previously reported findings. For example, these estimates using hospital discharge data are similar to those from an analysis of birth certificate data, which found that maternal HCV infection almost doubled during 2009–2014 from 1.8 to 3.4 per 1,000 live births (*2*). Increased likelihood of HCV infection, opioid use disorder diagnosis, or both among women with publicly billed deliveries is similar to previous findings that women with HCV infection were more likely to be Medicaid-insured (*4*). In this analysis, Native American women were significantly more likely to have an HCV infection or opioid use disorder diagnosis at delivery than were non-Hispanic black women. High rates of overdose deaths and HCV infection in American Indian and Alaska Native persons have been previously noted in the general adult population (*7*,*8*). Lower HCV infection rates at delivery among women in the West reflect distribution of HCV infection in the general population (*1*).

Current U.S. Preventive Service Task Force and CDC guidelines recommend hepatitis C testing for persons at high risk (e.g., persons who inject drugs^†^^,^^§^); however, epidemiologic changes in HCV infection in the United States have prompted a review of the evidence informing HCV testing by the U.S. Preventive Services Task Force and CDC. The American Association for the Study of Liver Diseases and the Infectious Diseases Society of America recommend hepatitis C screening for all pregnant women (*9*). Hepatitis C treatment for adults with direct-acting antiviral agents consists of an oral regimen of ≤12 weeks, resulting in a virologic cure in >90% of infected persons (*10*). Although treatment of HCV infection with direct-acting antiviral agents during pregnancy is not approved (*10*), testing remains important to identify infections, engage infected women in postpartum treatment, and identify infants who might have been exposed. Left untreated, HCV infection might lead to cirrhosis and pose continued risk to others through parenteral exposures (e.g., injection drug use or transmission via subsequent pregnancies) (*1*).

The findings in this report are subject to at least five limitations. First, this study likely produced underestimates of opioid use disorder and HCV infection. Although universal screening for substance use is the standard of care during pregnancy, it is not universally implemented. Further, stigma and associated fear of reporting opioid use disorder likely reduces self-disclosure. Risk-based hepatitis C testing is the current care standard but might not be adequately implemented. Second, increases in observed rates might reflect changes in screening practices and protocols for opioid use disorder and HCV in addition to actual increases in these conditions. Third, ICD-9-CM does not differentiate between chronic or incident acute HCV infection. Fourth, these analyses might not represent most recent trends because data were only analyzed up to the third quarter of 2015. Finally, results of this analysis are only generalizable to hospital births; however, fewer than 2% of U.S births occur outside of the hospital.^¶^

Opioid use disorder (*3*) and HCV infection rates significantly increased during 2000–2015 among women delivering in hospitals in the United States. HCV infection rates at delivery were significantly higher among women with opioid use disorder than among those who did not have opioid use disorder. Treatment of opioid use disorder should include screening and referral for related conditions such as HCV infection.

**Summary**

**What is already known about this topic?**

Ecological studies link increases in hepatitis C virus (HCV) infection to the U.S. opioid crisis. Opioid use disorder among pregnant women has increased; the majority of those with HCV infection have opioid use disorder.

**What is added by this report?**

The U.S. rate of HCV infection at delivery increased from 0.8 per 1,000 live births in 2000 to 4.1 in 2015, including increases from 87.4 to 216.9 and from 0.7 to 2.6 among women with and without opioid use disorder, respectively.

**What are the implications for public health practice?**

Treatment of opioid use disorder should include screening and referral for related conditions such as HCV infection.

## Footnotes

* ICD-9-CM codes related to opioid dependence and nondependent abuse, in remission, were included in this analysis because both early remission and opioid use disorder could have occurred during pregnancy.

† https://www.uspreventiveservicestaskforce.org/Page/Document/UpdateSummaryFinal/hepatitis-c-screening.

§ https://www.cdc.gov/hepatitis/hcv/guidelinesc.htm.

¶
https://www.cdc.gov/nchs/data/nvsr/nvsr66/nvsr66_01.pdf .
